# Embedding social prescribing in primary care in England and Scotland: a qualitative study of experiences, roles, challenges, and sustainability

**DOI:** 10.1016/j.lanprc.2026.100128

**Published:** 2026-04

**Authors:** Eddie Donaghy, Stewart W Mercer, Heather Brant, Hilllary Collins, Manal Etemadi, Yuri Hamashima, Hugh McLeod, Catherine A O'Donnell, Caroline Sanders, Mihirini Sirisena, Paul Wilson

**Affiliations:** a Usher Institute, University of Edinburgh, Edinburgh, UK; b Bristol Medical School, Population Health Sciences, University of Bristol, Bristol and National Institute for Health Research Applied Research Collaboration West (ARC West), Bristol, UK; c General Practice and Primary Care, School of Health & Wellbeing, University of Glasgow, Glasgow, UK; d Centre for Primary Care and Health Services Research, University of Manchester, Manchester, UK; e Population Health Sciences Institute, Faculty of Medical Sciences, Newcastle upon Tyne, UK

## Abstract

**Background:**

In the last decade, social prescribing through link workers based in general practice has become a major policy in the UK, but little is known about the implementation of this strategy. We aimed to explore the roles, challenges, and effects of social prescribing link workers (SPLWs) across different models of employment, organisation, and management in England and Scotland.

**Methods:**

In this qualitative study, we conducted semi-structured interviews with stakeholders in two regions in England (National Institute for Health and care Research [NIHR] Applied Research Collaboration [ARC] West and NIHR ARC North East and North Cumbria) and two in Scotland (National Health Service [NHS] Greater Glasgow & Clyde and NHS Lothian). Stakeholders were participants actively involved in SPLW activities and comprised patients aged 18 years or older receiving SPLW support; individual SPLWs; general practice staff who referred patients to the SPLW; leads of voluntary, community, and social enterprises (VCSEs) employing or hosting SPLWs; VCSE leads not employing or hosting SPLWs but supporting referred patients; and strategic leaders working with SPLW services. Purposive sampling was used to ensure diversity in patients’ age, gender, and socioeconomic deprivation (according to the Index of Multiple Deprivation in England or Sottish Index of Multiple Deprivation in Scotland) and variation in stakeholder roles. Interviews were continued until data saturation was reached. Transcribed interviews were analysed reflexively using inductive thematic analysis.

**Findings:**

Between Feb 16 and Nov 15, 2024, we conducted interviews with 130 stakeholders: 36 SPLWs, 28 patients, 28 referring professionals, ten VCSE leads hosting SPLWs, 14 VCSE leads not hosting SPLWs, and 14 strategic leads. Patient ages ranged from 18 years to older than 65 years; 16 (57%) patients were female, 12 (43%) were male, 26 (93%) were of White British or Scottish ethnicity, one (4%) was of south Asian ethnicity, and one (4%) was of mixed or multiple ethnicity. Six key themes emerged. The first was varied backgrounds, with SPLWs typically coming from the voluntary sector or NHS, driven by a desire to empower patients. The second was changing role, with SPLWs now seeing many more patients with highly complex needs, including the social determinants of health in deprived areas, than they did previously. The third theme was therapeutic relationship, whereby building empathic relationships was crucial but depended on SPLWs’ backgrounds, training, and support. The fourth theme, benefits of the SPLWs, was based on SPLWs helping patients to build confidence and increase their independence and, in general practitioners, reducing moral distress and possibly reducing workload. The fifth theme was employment models, organisation, and management, with integration into general practices facilitated by SPLWs working in one or two practices and with robust organisational support. For the final theme, challenges and sustainability, the challenges of the SPLW role included job retention, burnout, variable pay levels, and few opportunities for career progression; the sustainability of this role was threatened by short-term funding, increased service demand, and financial cuts to essential services.

**Interpretation:**

This study highlights the vital role of SPLWs in England and Scotland and the need for stable support and funding amid ongoing financial challenges in the statutory and voluntary sectors.

**Funding:**

National Institute for Health and Care Research.

## Introduction

Health services worldwide are facing increasing pressures due to ageing populations, increasing mental illness and multimorbidity, growing health-care costs,^1^^,^^2^ and shortages within the health-care workforce, particularly of general practitioners (GPs).^3^^,^^4^ England and Scotland also have growing health inequalities,^5^ exacerbated by a mismatch between patient need and health-care supply (the inverse care law).^6^

**Research in context**

**Evidence before this study**

We searched PubMed and Web of Science for systematic, scoping, and realist reviews (published between Jan 1, 2000, and Dec 31, 2025) using search terms such as “social prescribing”, “non-medical referral”, “community referral”, “link worker”, and “care navigator”. Systematic reviews have documented how individuals in the social prescribing link worker (SPLW) role can work with patients to support access and engagement with sources of support in the community and with health services more generally. Systematic reviews have also documented that, historically, the evidence base for social prescribing is suboptimal, making it difficult to judge how and in what circumstances SPLWs can deliver benefits.

**Added value of this study**

Using qualitative data drawn from four geographical areas, our findings emphasise that SPLWs are increasingly providing support regarding issues relating to the social determinants of health, especially in areas of high socioeconomic deprivation. Much of this activity is not captured routinely in primary care. Our findings highlight the importance of integrating SPLWs into primary care teams to ensure appropriate support for the role and delivery of joined up care for the patient.

**Implications of all the available evidence**

SPLWs play a central role in supporting patients with complex needs. The sustainability of the SPLW role is threatened by unstable funding, increasing service demands due to ongoing economic austerity, and worsening access to statutory and non-statutory services. Further exploration of the long-term influence of SPLWs on health inequalities and their role in prevention efforts across different settings and contexts is warranted.

Consequently, transforming primary care has become a policy imperative. Together with the expansion of primary care teams,^7^ social prescribing has become a key policy strategy to help address current primary care challenges and reduce health inequalities.^8^^,^^9^ Over the past decade, policies have been introduced in England^10^ and Scotland^11^ to introduce social prescribing through link workers based in general practice. These social prescribing link workers (SPLWs) aim to empower patients to improve their own health by linking them to local voluntary, community, and social enterprise (VCSE) organisations.^12^ The deployment of SPLWs at scale began in 2019 in England and in 2017 in Scotland; there were approximately 3500 SPLWs in England in 2023^13^ and 340 in Scotland in 2025.^14^

Although SPLWs aim to help support patients, their long-term influence on health inequalities—a key policy aim in both countries—remains unclear.^15^, ^16^, ^17^ Additionally, the evidence base for the effectiveness of social prescribing—although growing—remains insufficient and inconclusive.^18^ Part of the challenge lies in the different ways in which SPLWs are embedded into existing primary care systems, with variation in who employs SPLWs and who these link workers are accountable to. To understand the role of SPLWs in a UK context, we conducted a large, multimethods programme of research on SPLWs. Our quantitative analyses using national datasets relating to population health outcomes and GP workload will be reported elsewhere. In this Article, we report on the findings of qualitative interviews with key stakeholders. Using in-depth qualitative research, we aimed to explore the roles of SPLWs, challenges, and effects across different models of employment, organisation, and management of SPLWs in England and Scotland. Our analysis took a reflexive inductive approach to identify key themes, followed by a deductive framework approach to systematically explore the relationships between themes and the factors affecting them.

## Methods

### Study design

This qualitative study is part of a larger multisite, mixed-methods study evaluating the roll-out of SPLWs across primary care in England and Scotland.^19^ Here, we explored the experiences of key stakeholders involved with SPLW services from eight case study sites across four research regions in England and Scotland, and we report on the qualitative findings; quantitative findings are being published separately.

Four regions participated in the qualitative component of the study: two in England (National Institute for Health and care Research [NIHR] Applied Research Collaboration [ARC] North East and North Cumbria and NIHR ARC West) and two in Scotland (National Health Service [NHS] Greater Glasgow & Clyde and NHS Lothian). These regions vary in terms of geography, population size, ethnic diversity, demographic characteristics, socioeconomic deprivation, rurality, and distance to services. Details of the geographical footprints and the organisation and delivery of health and care services in these four regions are in panel 1. Our previous mapping of SPLW services across the participating research regions^20^ facilitated the identification of suitable case study sites. There were three case study sites in the NIHR ARC North East and North Cumbria region, three in NIHR ARC West region, one in NHS Greater Glasgow & Clyde region, and one in NHS Lothian region. Using a maximum variation design, these case study sites reflected differences in SPLW service model delivery, individual and area characteristics, socioeconomic deprivation levels (according to the Index of Multiple Deprivation in England or Scottish Index of Multiple Deprivation in Scotland), urban versus rural settings, and availability of community assets and activities. Individual case study sites remain unnamed to protect participant confidentiality, but we provide additional information on each case site in terms of urbanicity, rurality, deprivation, and ethnic diversity in the appendix (p 2).

Panel 1 **Research regions**

**National Institute for Health and Social Care (NIHR) Applied Research Collaboration (ARC) North East and North Cumbria**

• One of 15 ARCs across England supporting applied health and care research that responds to and meets the needs of the local health and care system
• Encompasses urban and rural populations including Newcastle, Gateshead, North and South Tyneside, Northumberland, and North Cumbria
• Organisation and delivery of health and care services is by the North East and North Cumbria Integrated Care Partnership
• Social prescribing link worker (SPLW) service provision is mostly outsourced to existing voluntary, community, and social enterprises (VCSEs), but management input regarding SPLW training, support, and oversight is retained; some SPLWs are directly employed and managed in primary care
• Population is approximately 3·2 million

**NIHR ARC West**

• One of 15 ARCs across England supporting applied health and care research that responds to and meets the needs of the local health and care system
• Encompasses urban and rural populations including Bristol, North Somerset, South Gloucestershire, Gloucestershire, Bath, North East Somerset, Swindon, and Wiltshire
• Health and care services are organised and delivered by three Integrated Care Systems: Bristol, North Somerset and South Gloucestershire Healthier Together, One Gloucestershire, and Bath and North East Somerset, Swindon and Wiltshire Together
• SPLW service provision is mostly outsourced to existing VCSEs, but management input regarding SPLW training, support, and oversight is retained; some SPLWs are directly employed and managed in primary care
• Population is approximately 2·7 million

**National Health Service (NHS) Greater Glasgow & Clyde**

• Encompasses mostly urban populations including Glasgow, Dunbartonshire, Renfrewshire, and Inverclyde
• Organisation and delivery of health and care services is by NHS Greater Glasgow & Clyde
• SPLW service provision is mostly outsourced to existing VCSEs, but management input regarding SPLW training, support, and oversight is retained
• Population is approximately 2·7 million

**NHS Lothian**

• Encompasses mostly urban populations including Edinburgh, Midlothian, East Lothian, and West Lothian
• Organisation and delivery of health and care services is by NHS Lothian
• SPLW service provision is mostly outsourced to existing VCSEs, but management input regarding SPLW training, support, and oversight is retained
• Population is approximately 900 000

The protocol for the study is available online.^19^ There were two protocol amendments during the study. First, we included interviews with strategic leads involved in SPLW delivery across the four regions as we considered it important to provide strategic-level insight into the implementation and roll-out of this service within primary care provided by the NHS in England and Scotland; this amendment was accepted by the funder on Oct 5, 2022. Second, recruitment was halted at 130 participants, rather the initial target of 160, as during the data collection and analysis process it became clear that data saturation had been reached in each group; this amendment was accepted by the funder on Dec 1, 2024. Ethical approval for this study was granted by North East-York Research Ethics Committee (reference 23/NE/0176O). Verbal informed consent was audio-recorded before telephone or online interviews, with written consent obtained for a small number of face-to-face interviews.

### Participants

The previous mapping of SPLW services^20^ introduced local researchers (ED, HC, ME, HB, and MS) to key SPLW leads, who then introduced the study at the eight case study sites. Eligible participants were stakeholders actively engaged in SPLW activities: patients aged 18 years and older receiving SPLW support; individual SPLWs; general practice staff who referred patients to the SPLW (referring professionals); leads of VCSEs employing or hosting SPLWs; VCSE leads not employing or hosting SPLWs but supporting referred patients; and strategic leads working with SPLW services. Lacking the capacity to consent was the only exclusion criterion. Purposive sampling ensured representation of key roles and experiences across SPLW services, general practices, VCSE organisations, and patients. The criteria used for sampling were time in post (for SPLWs); variation in age, gender, socioeconomic deprivation, multimorbidity, intensity of SPLW engagement, and inclusion of harder-to-reach individuals (for patients); variation in primary care experience (for referring professionals); organisational role, size of organisation (local community, regional, or national), financial resources, full-time and part-time staff capacity, and system position (ie, involvement within the local health and social care system; for VCSE leads); and experience in system-level oversight (for strategic leads). Participating SPLWs identified current or recent (ie, seen in the previous 3–4 months) patients for the study, and, after obtaining patient consent, shared their contact details with local researchers. Details of the number and type of participants recruited by site and the researcher responsible for recruitment in each site are provided in the appendix (pp 3–4). Participants received a consent form by email before interview.

### Data collection

Data were collected through in-depth, semi-structured, one-to-one interviews, with most interviews being conducted via telephone or Microsoft Teams and fewer than 10% being conducted face-to-face (in a secured room in a community setting). Six separate semi-structured interview guides were developed, one per stakeholder group (appendix p 5). A qualitative data analysis group was established, consisting of the study coauthors. The topic guides were developed by ED, SWM, CS, and CAO and then refined by local study researchers (coauthors ED, HC, ME, HB, YH, and MS). After the first two or three interviews with each of the six SPLW stakeholders, the topic guides were reviewed by the same coauthors, and appropriate changes made following discussions.

The coauthors conducting the qualitative interviews (four self-identified their sex or gender as female, one as male, and one as non-binary) were all experienced in qualitative health research. For ARC West, interviews were done by three coauthors: HB (Research Fellow at the University of Bristol at the time of the study), ME (Research Fellow at the University of Bristol at the time of the study), and YH (Senior Research Associate at the University of Bristol at the time of the study), all three of whom are experienced in interview-based studies and thematic analysis and trained in the use of NVivo software to conduct qualitative analysis. For ARC North East and North Cumbria, interviews were done by MS (Research Associate at Newcastle University at the time of the study; experienced in qualitative research, including interview-based studies and thematic analysis, and trained in the use of NVivo software for qualitative analysis). For NHS Lothian, interviews were done by ED (Research Fellow at the University of Edinburgh at the time of the study; trained in interview-based methods, thematic analysis, and NVivo). For NHS Greater Glasgow & Clyde, interviews were done by HC (Research Associate at the University of Glasgow at the time of the study; experienced in interview-based studies, thematic analysis, and use of NVivo software and trained in a range of qualitative methods). Apart from the interviewers for ARC West (ME, HB, and YH), interviewers across the four regions had no previous relationships with each other before the study. Regular monthly meetings at the start of the mixed-methods project, focused on mapping regional SPLW services, meant that by the time the qualitative phase of the study began, the interviewers were personally and professionally acquainted. Further qualitative monthly meetings for topic guide development, participant recruitment, and data analysis strengthened team cohesion and a shared research ethos.

Before interview, participants received an invitation email (or a letter, for some patients) and a participant information sheet outlining the purpose of the research. The invitation email or letter explained who the individual interviewers were (in terms of their academic positions) and invited participants to ask questions—by email or verbally (by telephone or via Microsoft Teams)—just before the interview began and before participants had given their consent to take part in the study. Interviews were anticipated to last up to 1 h. Participants did not have any information about the researchers other than their name, formal title, job position and where they were employed. At the start of each interview, the interviewer briefly re-introduced themselves and restated the purpose of the study.

For the interviews, topic guides specific to each of the six stakeholders were used. No interviews were required to be translated, and no repeat interviews were conducted. Pilot interviews were not conducted due to the experience of the team in qualitative research and the timelines involved. All interviews were audio-recorded and transcribed verbatim without the use of AI or automated tools (by 1st Class Secretarial Services; Gorebridge, Scotland) and were anonymised by assigning each participant a unique reference number and a pseudonym for attributable quotes. All interview transcripts were checked for accuracy against the original audio recordings by the individual researcher who conducted the interviews, providing internal verification. All local study researchers also took field notes immediately after the interviews had ended, which were discussed at monthly qualitative data analysis meetings. Due to time constraints, interview transcripts were not returned to participants for comment or correction.

Demographic data were self-reported by patient participants before interview with predefined categories (age [18–29 years, 30–49 years, 50–65 years, or >65 years], gender [male, female, non-binary, or prefer not to say], ethnicity [White, mixed or multiple ethnicity, and multiple options pertaining to Asian, Pakistani, Bangladeshi, Chinese, African, Caribbean or Black Caribbean, and Arab ethnicities], postcode, living arrangements [lives alone: yes, no], and multimorbidity [more than one long-term condition: yes, no]). No demographic data were collected from other stakeholder groups. Individuals were informed that their involvement in this study was voluntary and that they could withdraw participation at any time.

### Data analysis

ED, HC, HB, ME, and MS selected one representative transcript from each of the six stakeholder groups in their respective regions (n=30) for the initial round of coding. Nominated transcripts for each stakeholder group were then independently coded by these investigators. Initial coding interpretations were circulated between investigators, and interpretations of each stakeholder group’s coding were discussed at a monthly meeting. Coding frames were iteratively refined until consensus was reached on each of the six separate coding frames. All interview transcripts were then coded using QSR NVivo 12 and QSR NVivo 14 (coding tree provided in the appendix [pp 6–8]).

Data were analysed inductively using a thematic approach by ED, HC, HB, ME, MS, and YH on the basis of Braun and Clarke’s six-phase framework.^21^^,^^22^ Researchers familiarised themselves with transcripts, generated codes, and organised these into initial themes, which were discussed, reviewed, defined, and named. A reflexive approach was maintained to acknowledge the researchers’ active role in these interpretations. Named themes were agreed on, circulated, and further discussed at an additional wider data analysis meeting involving SWM, PW, CAO, CS, and HM, focusing on key findings and further theme development. Initial themes were also shared and discussed with members of the Patient and Public Involvement and Engagement group, who were a core part of the research team for this study and provided valuable feedback. The Patient and Public Involvement and Engagement group comprised members who were drawn from the study regions and brought a range of skills, knowledge, voluntary experience, and lived experience to ensure that a diverse public voice informed our research and methods. One member was also involved in the original research application bid, and at least one member attended all research management group meetings. Group members were involved throughout the study and provided input into study planning, data collection, and quantitative and qualitative analysis.

Analytical methods were aligned with the sampling strategy by initially analysing interview data separately within each stakeholder group, consistent with the purposive sampling approach. Themes were then compared and integrated across stakeholder groups to identify shared and contrasting perspectives. Potential bias was addressed through these reflexive team discussions, the shared standardised interview guides, consistent procedures, collaborative coding, and team-based analysis, with interpretations reviewed at the monthly meetings (a reflexivity statement is provided in the appendix [p 9]).

To further explore relationships between themes, we conducted a post-hoc analysis on the SPLW interviews using a deductive framework approach based on the method of Spencer and Ritchie.^23^ This analysis involved the compilation of a matrix with a summary of each theme for each of the 36 SPLW interviews, facilitating an in-depth analysis of the relationships between themes and factors associated with their variation.^24^

### Role of the funding source

The funder of the study had no role in study design, data collection, data analysis, data interpretation, or writing of the report.

## Results

Between Feb 16 and Nov 15, 2024, we approached 160 stakeholders across all six SPLW stakeholder groups and conducted interviews with 130 (81%) stakeholders: 36 SPLWs, 28 patients, 28 referring professionals, ten VCSE leads hosting SPLWs, 14 VCSE leads not hosting SPLWs, and 14 strategic leads. Numbers of each type of stakeholder approached, numbers of those refusing the invitation to participate, and reasons for refusal in each stakeholder group are provided in the appendix (p 10). Interview length ranged from 19 min to 94 min, with a median length of 49 min (IQR 37–56). Time in post for SPLWs ranged from 3 months to 7 years (appendix p 11), with a median duration of 24 months (IQR 12–48). 20 (71%) of 28 participating patients lived in areas of high socioeconomic deprivation, and 18 (64%) had more than one long-term condition (table). 16 (57%) patients were female and 12 (43%) were male; 26 (93%) were of White British or Scottish ethnicity, one (4%) was of south Asian ethnicity, and one (4%) was of mixed or multiple ethnicity. Of the 28 referring professionals, most (18 [64%]) were GPs (appendix [p 11]).

**Table:** Demographic characteristics of patients

	Patient participants (N=28)
**Age range, years**	
18–29	2 (7%)
30–49	10 (36%)
50–65	8 (29%)
>65	8 (29%)
**Gender**	
Female	16 (57%)
Male	12 (43%)
Non-binary	0
Prefer not to say	0
**Ethnicity**	
White	26 (93%)
South Asian	1 (4%)
Mixed or multiple ethnicity	1 (4%)
Other∗	0
**Deprivation status**†	
High	20 (71%)
Low or medium	8 (29%)
**Employment status**	
Unemployed	15 (54%)
Employed	6 (21%)
Retired	7 (25%)
**Lives alone**	
Yes	13 (46%)
No	15 (54%)
**Has more than one long-term condition**	
Yes	18 (64%)
No	10 (36%)
**Number of sessions with SPLW**	
Two	1 (4%)
Three	2 (7%)
Four or more	25 (89%)

Data are n (%). Summed percentages might exceed 100% because of rounding. SPLW=social prescribing link worker.

∗ Other ethnicities provided as options in the questionnaire included Pakistani, Bangladeshi, Chinese, African, Caribbean or Black Caribbean, Black, and Arab (and permutations thereof in terms of British or Scottish).

† Based on the Index of Multiple Deprivation in England and Scottish Index of Multiple Deprivation in Scotland.

Each quote includes the unique reference number of the participant, the region, and (where applicable) the case site number. Six major themes emerged from the analysis: (1) varied backgrounds; (2) changing role; (3) therapeutic relationship; (4) benefits of the SPLWs; (5) employment models, organisation, and management; and (6) challenges and sustainability. Quotes in panel 2 reflect participants’ views and experiences as reported in these themes and subthemes (additional quotes are provided in the appendix [pp 13–22]).

Panel 2 **Major themes**

**Theme one: varied backgrounds of social prescribing link workers (SPLWs)**

“Probably about a third [of SPLWs] are people who have got really solid and transferrable experience. People who have either come from a social work, third sector homelessness or community health organisation background. We get some people from nursing or occupational therapy background.” (VCSE Hosting 1; Lothian)

“I used to be a community support worker, a link between mental health and the community. Working with community groups, working with stigma of mental health, delivering workshops. I had mental health first aid training. Working with community members to upskill them and empower them. I’m also an adult education teacher, a therapist, a holistic therapist and a yoga teacher. I have lots of different skills.” (SPLW3; West of England, case site 3)

“It’s definitely a field that I’m quite keen and quite drawn to, certainly supporting people…[host VCSE]…they’re very keen on people who are just absolutely dedicated to supporting people and empathy and so on. So, it was great.” (SPLW2; West of England, case site 1)

**Theme two: changing role of SPLWs**

“A lot of people have quite severe adverse events from childhood, so sexual abuse or neglect, physical abuse in childhood…because of the abuse that has happened, they’ve gone on a path of using coping strategies that are quite negative—alcohol, drug use. So, I have quite a lot of those cases. I think probably from COVID, it’s got worse…So, I’m trying to learn how to set boundaries and think, this is going too far from what I can actually do.” (SPLW3; Greater Glasgow & Clyde)

“Initially, I thought that’s what we were there for, to get people into the community, get them into groups, get them to socialise, get them out of the house. I thought that was what the social prescriber was. But social prescribing is totally the wrong name for our job…. A lot of what we do is not social prescribing, its benefits, its housing, it’s equipment. It’s stuff like that.” (SPLW3; North East and North Cumbria, case site 2)

“I think the stereotype is the role is you’re taking people to knitting groups and things like that, but that’s just not the reality. I’d say about 10% of cases are around social activities. I do a lot of phone calls about housing or you attend their PIP [disability benefits] assessment…so you’re chipping in from the sides and advocating.” (SPLW2; North East and North Cumbria, case site 1)

“…at the point of contact, and meeting the links worker, myself and my children were facing homelessness…And the links worker put a lot of calls out to get agencies involved. Because I have a disabled child, as well, the links worker put calls out to a carer group that I didn’t know that I could attend, to give me support. Also, they put me in contact with [financial organisation], who were able to go over benefits, to see what other things I would be entitled to.” (Patient 1; Greater Glasgow & Clyde)

“The council budgets have been squeezed and squeezed and squeezed…We have people walking in, in crisis. I can phone the intensive mental health team. They’ll tell me to ring another mental health team. They’ll tell me to ring another mental health team. They’ll tell me to ring an ambulance. They’ll tell me to ring the police, they’ll tell me to ring the doctors. And nobody comes. Nobody comes at the end…And we can just go round and round in circles. The system is under enormous pressure…Many more people are experiencing crisis.” (Strategic Lead 1; West of England, case site 2)

“Ninety per cent of the patients they [SPLWs] see have significant mental health worries, whether they present with a mental health concern as a primary need or whether that is additional to a financial worry or housing or physical health worry. So, in terms of challenges, they [SPLWs] are covering lots of different gaps in the system. With waiting lists for mental health services being so long or the threshold being so high, I think they [SPLWs] find themselves in roles that they are not necessarily trained for.” (Strategic Lead 3; West of England, case site 2)

“As the state seems to be withdrawing, or putting barriers up to entry, we keep asking ourselves— well what’s our [VCSE] role in this? We don’t want to become a mini statutory sector organisation, but as the state withdraws, more and more gaps are opening up…” (VCSE Hosting 1; West of England, case site 3)

“We’re working with partners, particularly the local authority, who are very stretched. A lot of people are coming to us with housing problems, struggling to access welfare benefits, don’t have enough food…If you were working with a very highly functioning local authority where there’s stable social work; if you’re working with a housing provider where there’s plenty of accommodation and it’s suitable; if you’re working with a DWP [Department of Work and Pensions] who are easy to access and encouraging people to claim the benefits that they’re entitled to—it would be a very different picture… people’s health suffers because those structures are not as good as they once were.” (SPLW5; West of England, case site 2)

**Theme three: therapeutic relationship**

“I think you need time…for them to actually open up and talk to you. It’s quite emotional. It’s emotional for them to tell you what’s going on in their lives and it’s a hard thing for them. So, first of all, you’ve got your trust to establish with them and make sure they’re comfortable with you.” (SPLW1; Lothian)

“I think at that first appointment just listening and showing them that I’m empathetic and I’m listening to what they’re saying and I’m understanding what their situation is. Just building trust up in that way and being transparent with them from the start.” (SPLW5; North East and North Cumbria, case site 2)

“We are dealing with people who come with all sorts of trauma…We have regular conversations with people about suicide, about abuse, historical abuse. All of the things that come with living in poverty. People come in with the weight of the world on them and then they are, like, ‘My god, I feel seen, I feel heard, I feel better’. I am here to listen. I do feel that that, for a lot of people, is enough.” (SPLW4; Lothian)

“I deeply regret we have so little time as GPs [general practitioners]…Time is important because most of our patients have very complex lives…Our link worker is skilled at relationships, has a real understanding of the barriers that patients face. And they also have more time…I could do with five link workers quite frankly.” (Referring Professional 5; Lothian)

“I suppose it was comforting, very easy to talk to, which I think is the main thing…But very easy, very relaxing…they made you feel at ease, that’s the main thing…there was no embarrassment or anything…you almost felt you made new friends and they were just very easy to talk to.” (Patient 1; West of England, case site 3)

“It’s literally just having somebody like [the SPLW] to talk to. What I quite like about my link worker is that, she’s also a normal, everyday person. She’s not a therapist or a doctor. So, when you’re talking to her, it’s like you’re talking to a friend rather than somebody in a strictly professional capacity—it can take a weight off your shoulders.” (Patient 2; West of England, case site 1)

**Theme four: benefits of SPLWs**

***To service users***

“I’ve struggled with anxiety and depression for years, I’m medicated for it, I was feeling lonely. There is a lot of behavioural issues with my brother. I’m his full-time carer. I just thought, I don’t know what to do with myself. So, I met [SPLW]. Financially I couldn’t join a private gym, he managed to refer me for a CAP [Community Access Programme] card. So, that got me back in the gym and swimming—a godsend…through a charity I got access to a trained counsellor who I’m seeing once a week. There’s another charity, he got me funding to get a wee break which I did and that was a game-changer. That was the first time that I’ve been hopeful about my future…it’s changed everything, I’m trying to make plans.” (Patient 5; Lothian)

“Well, my anxiety is a lot better. I started seeing [SPLW]. She got me out to do the farm thing…I’d be doing things on the farm, which was really nice…then I started going along to the men’s breakfast and doing all that and going on trips. So, I think the process of doing all that impacted my wellbeing to the fact where my state of mind has enabled me to get in a relationship. I started working out. Now I’m actually going to be going to college in October.” (Patient 2; West of England, case site 3)

***To general practitioners (GPs)***

“Well, [as a GP] I actually don't think I can work without a links worker now. Not without that moral thing, is it moral distress?…I remember people sitting in front of me in real distress, both of us distressed, because me feeling—I can't really help you, and I feel so awful…I have less of them when I've got a links worker that’s working effectively…..I can probably do more constructive work—it sounds awful—if my morning isn't filled with people who are utterly overwhelmed by their situation.” (Referring Professional 2; Greater Glasgow & Clyde)

“One of the things that’s critical to this is that what a links worker does—it keeps me working [as a GP] for a start. Honestly. My alternative to giving these patients to the link workers would have been to ignore them in a callous way, which is emotionally very difficult for me but it would have been the only way for me to survive. Or to [prescribe] drugs for them…So that would have been the alternative and it just goes against all my ethics, so I would have been tortured.” (Referring Professional 5; Greater Glasgow & Clyde)

“Oh, 100 percent. Yeah, 100 percent [workload has been reduced]. Certain patients that sometimes just need that contact. There’s two I can think of off the top of my head, and the social prescribers are working very closely with each other. It really has reduced the amount of times the patients have seen me.” (Referring Professional 2; West of England, case site 2)

“It’s made my role as a GP more rewarding. Just knowing that you would link somebody who had been able to practically support this person with their debt was just enormously satisfying…but I would be amazed if there were a large number of GPs who felt that their overwhelming workload has been impacted by SPLWs, because when your head is under water, it doesn’t matter whether it’s 6 inches or 12 inches under the waterline, they [GPs] still absolutely, as a profession, feel under the waterline.” (Referring Professional 3; North East and North Cumbria, case site 2)

“Certainly, at my first practice, I felt the social prescriber definitely made a difference [workload-wise]…In my second one [practice], I didn’t find the social prescribers quite so helpful. I often found it was quite a quick signpost and that was it. They weren’t typically working with patients over a longer period, I didn’t feel they really got to know the patients. There wasn’t that same kind of relational continuity.” (Referring Professional 1: West of England, case site 3)

**Theme five: employment models, organisation, and management of SPLWs**

“Being more part of a team. Because it can be really isolating…So I think something to help people [SPLWs] to feel part of things…like some recognition of the work that people [SPLWs] are doing, because often it feels like you’re just chipping away doing things and there’s nobody really acknowledges what you’ve done…I think more could be done to help people [SPLWs] to feel included and valued really.” (SPLW5; North East and North Cumbria, case site 2)

“I think the clinicians know who their social prescribers are, they have a good rapport…They have a room on a regular basis at the surgery. We encourage them to be part of the practice team, so they go to coffee within the practice teams, they’re included in any social events and things. So they are part of the team and they’re not separated out. It does make a significant difference.” (Referring Professional 1; West of England, case site 1)

“I mean, they [SPLW] are not a member of the core team, because what they’re employed to do is actually signpost people away from primary care, into more appropriate services. They come and work here as an individual service, and when they’re here it’s one afternoon a week, so they’re not going to be part of the team in the same way, coming to lunch, and coming to all the meetings. No, they don’t come to our practice meetings. Because they’re spread too thinly to do that, they’re covering an enormous number of patients in our PCN [primary care network].” (Referring Professional 1; West of England, case site 3)

**Theme six: challenges and sustainability**

***Retention***

“I think it’s not sustainable at the moment because in terms of a career, it’s low paid, there’s high stress, there’s issues that are really hard, like hard-hitting things that you’re dealing with every day, and I think people are putting more and more onto link workers as well.” (SPLW3; Greater Glasgow & Clyde)

***Funding***

“Sustaining the funding is the thing. The biggest thing is trying to find mainstream funding for SPLWs that is not subject to political change. The reality is, unless there is some sort of baseline budget, then it is only ever funded for a period of time, unless they baseline it or mainstream it. It is funded for a political cycle or for a year at a time. That is really, really challenging, really challenging.” (Strategic Lead 1; Greater Glasgow & Clyde)

“Funding is the biggest challenge at the moment. We’ve just had to let the contract go and we were doing some great work and the PCN [primary care network] were really pleased with the work that we were doing. However, the financial viability of that contract meant that we were running at a significant loss and it comes back down to this thing about the management costs not being included in the funding…They don’t cover everything that needs to be covered.” (Strategic Lead 1; North East and North Cumbria, case site 3)

Regarding the first theme, SPLWs had diverse experience in health, social care, and VCSEs, which often supported their understanding of working with people with diverse needs and backgrounds. There were many reasons why they were attracted to becoming SPLWs, but all had a strong commitment to empowering patients to improve their wellbeing.

For the second theme (changing role of SPLWs), interview accounts highlighted the diversity of SPLWs’ caseloads. Some patients only required simple linking to appropriate community resources (reflecting the traditional social prescribing role), but, in recent years, there has been a substantial increase in individuals who require much more intensive ongoing support, especially in areas of socioeconomic deprivation. The complex needs of these patients commonly encompass social and financial aspects, social isolation, mental health issues, addiction, and multiple long-term conditions (multimorbidity). Relatedly, all interviewee stakeholder groups reported that a growing proportion of the workload of SPLWs concerned the basic social determinants of health, such as housing, heating, welfare benefits, and food supply. This increased complexity of workload presented practical and emotional challenges for SPLWs, with some feeling that they could not offer sufficient support.

The third theme covered the importance of the therapeutic relationship, with all respondents emphasising the core role of the SPLW in fostering good communication with patients to build a trusting relationship. SPLWs stressed the importance of having sufficient time with patients (often up to 1 h), especially with those with complex needs. Empathy, listening, and showing understanding were seen as key to being an effective SPLW. Most GPs understood the importance of time in developing a therapeutic relationship with patients and appreciated the fact that SPLWs had more time than they did with patients. Patients reported that being listened to in a non-judgemental way by the SPLW was very beneficial. For those with complex psychosocial problems who were regularly meeting the SPLW, this aspect was often as important as being signposted to a community group, or even more important.

Regarding theme four, the benefits of SPLWs to patients and GPs, many patients reported that the SPLW helped them build their confidence, increase their independence, and foster and strengthen relationships through community engagement, aiding their personal growth and fulfilment. Facilitators to patients’ ability to engage with suggested community resources included patients’ ability to access the resource, alignments with interests, and relationship with or trust in the SPLW. Many GPs reported the benefits of having a SPLW in the practice, including a reduction in their own stress levels and moral distress at not being able to provide the care they wanted to, especially for patients with complex needs living in under-resourced areas. GPs' views on whether their actual workload had been reduced were, however, mixed.

Theme five covered the employment, organisation, and management of SPLWs. Service delivery models varied between and within case study sites, with differing arrangements for the employment, salary, organisation, and management of the SPLWs. Irrespective of the different employment models, all SPLWs were allocated to one or more general practices, but the number of practices they covered varied widely, from one to seven. This variation led to challenges related to how SPLWs were viewed within the general practices. While some referring professionals viewed SPLWs as integral members of the team, other practices saw the role as an adjunct to the practice team, which adversely affected SPLWs’ job satisfaction.

The challenges covered by theme six included job retention, burnout, variable pay levels, and poor career progression. Moreover, the sustainability of the SPLW role is threatened by short-term funding, increased service demand, and financial cuts to essential services.

The relationships between these key themes and their interactions with micro-level, meso-level, and macro-level factors were explored in post-hoc analysis of the SPLW interviews to give a deeper understanding of the facilitators of effective SPLW working. Interpreting the themes through these multiple levels of factors draws out a more detailed analysis of some of the contradictions and complexities evident in the accounts.

At the micro level, there were clear tensions between the SPLWs’ desire to help patients (based on their commitment to empowerment) and the constraints imposed by their skills and previous training (eg, not trained in counselling), caseload, and the patients’ situation and needs. Although all SPLWs agreed on the importance of empathy and the development of a therapeutic relationship with patients, it was common for SPLWs to establish a professional boundary from the start for patients with complex needs, by explaining that they were not there as a therapist or counsellor.

“I think people get a bit confused like I’m not a support worker, I’m not a social worker, I’m not a counsellor. So, I try and be really clear with people about what my job is. Because I think that one of the things is not building up dependency on the service.”

SPLW2; Greater Glasgow & Clyde

The extent to which SPLWs were happy to work in depth with such patients was somewhat influenced by their professional background (eg, the few who had been social workers before becoming SPLWs were generally very comfortable working with such complexity) but was influenced more by the quality of their training and the level of formal mental health and other support they had in post. Similar factors influenced how comfortable SPLWs were in helping patients with the social determinants of health. However, this empathic approach could bring challenges. For example, one SPLW who had no previous background in working with people with complex problems, found the emotional toll initially overwhelming, requiring one-to-one counselling to help her cope with the demands of the job.

“I had a series of really emotional and difficult cases. I was offered, and I did see a counsellor. It can be very difficult, when people are talking about their past history of sexual abuse. The role can be hard. I actually saw this patient, he was feeling suicidal. He’d been cutting himself really badly for the week beforehand. I was with him for 3 hours, just trying to get support. He was in a really bad way.”

SPLW2; West of England, case site 3

At the meso level, factors that strongly enhanced the SPLWs’ motivation and job satisfaction included the SPLWs’ autonomy and feelings of competence, the amount and quality of SPLW training, support for the SPLWs’ mental health, and their integration or sense of belonging to a team. Integration and support from GPs and primary care staff in the practice seemed particularly valued, although good support from the VCSE, primary care network (in England), health and social care partnership (in Scotland), or SPLW network could, to some extent, substitute for a paucity of integration and support from general practices. Unsurprisingly, integration with general practices was related to the number of practices the SPLW had to cover and the availability of a room in the practice for the SPLWs. However, the attitude of the GPs to the concept of social prescribing and to the SPLW being seen as a member of the practice team varied within and between practices, often depending on how many practices or practice sites the SPLW covered.

“I don’t know them at all if I’m honest, and I think I am conscious that may be a symptom of such a big practice. So, we have four sites, and therefore we are a bit of a cog in a system sometimes. So, that might be a slight symptom of that, but I do think it could be a barrier, or at least it makes it slightly harder because you don’t have those dynamic conversations of ‘could I catch you about said person’ and communication…I think there would be benefit from a bit more face to face, I find it difficult to see the strong effects of social prescribers.”

Referring Professional 1; West of England, case site 3

A number of stakeholders also raised concerns that SPLWs and VCSE organisations were now receiving referrals that were not within the original scope and remit of social prescribing (ie, linking patients to community resources) and what SPLWs can safely offer based on their training and skill sets. This situation was due, in part, to statutory services such as mental health services, welfare benefits, and housing services reportedly providing less support than in the past, leaving gaps in statutory NHS and social services that SPLWs and VCSE organisations increasingly feel pressured to try to fill.

At the macro level, the policies and practices implemented by the employing organisation also impinged on the SPLW role. For example, some organisations set throughput targets for the number of patients seen by the SPLWs, which, if high, deterred them from working in depth with patients with more complex issues. Caseload number varied widely between SPLWs across sites and regions, influencing the amount of time and emotional support that SPLWs could give to patients with complex needs. However, it was often left to the individual SPLW to navigate and address this tension between need and numbers, balancing the emotional labour inherent in dealing with patients with complex health and social needs relating to poverty.

No particular employment model emerged as being clearly superior, but good clinical supervision and support from GPs, with a supportive VCSE and primary care network or health and social care partnership who all communicated with each other, were particularly valued by SPLWs. Role definition in some regions (ie, exactly what a SPLW should be doing and not doing) was often lacking or blurred, which presented clear challenges.

“It felt very unbounded, and I don’t think it’s very good for your mental health, for your workers’ stress levels when you end up feeling responsible for all of these things in any patient’s life.”

SPLW2; North East and North Cumbria, case site 1

Although they were highly committed, many SPLWs had concerns over job retention related to factors such as burnout, low pay, lack of job security, lack of career structure and progression, insufficient training, support and clinical supervision, stress, and isolation. Regional and site variation in terms of the various dimensions of support was apparent. SPLWs appeared less happy in their roles in two regions, reporting less satisfaction with training and less support from general practices and other SPLWs. As a result, compared with SPLWs in the other two regions, they felt less competent in their role, were unhappier in dealing with patients with complex needs and/or those needing support with the basic social determinants of health, and were more likely to feel that the SPLW role was not sustainable in its present form. The sustainability of the SPLW role was in question across all regions. Concerns were raised about the absence of standardisation of the SPLW roles, the increasing referrals concerning patients’ basic socioeconomic needs, and risk management issues relating to the increasing complexity of patients being referred to SPLWs with regard to mental health problems.

“So, standard operating procedures, standard process…there's a lot of variation in terms of how social prescribing is delivered. And just really simple things like the level of risk that is appropriate for social prescribing to take on…because I don’t think we’ve even got a clear definition of what social prescribing is and exactly who it works with?”

Strategic Lead 1; West of England, case site 3

“I have to be very careful how much we say yes to. Not to open those floodgates. Keep it quite a managed role. We don’t want anyone working outside their capacity or their experience, or qualifications. I think we’re getting into that territory a bit with mental health now. I am very aware that some of the people that we see probably are way above where we [as a SPLW service] are supposed to align.”

Strategic Lead 1; North East and North Cumbria, case site 2

The ongoing effects of cuts in public funding for NHS, social statutory, and VCSE funding were also a key theme throughout all regions and were mentioned by all GPs and strategic leads interviewed.

“Yeah, charities, public services, they’re all overwhelmed and oversubscribed, massive waiting lists, no funds or having to shut their doors because they’ve got too much work already. Yeah, I haven’t encountered any service that doesn’t seem to be struggling.”

SPLW2; West of England, case site 3

“There is a lot of tardiness and people don’t get called back or feel dismissed. There’s definitely a lot of advocacy, acting on behalf of patients, where they feel that the systems are not listening or responding to them.”

SPLW2; Lothian

In England, several interviewees suggested that social prescribing would be better used to focus on the geographical areas with greater socioeconomic deprivation rather than operating as a universal service. A number of strategic leads in Scotland believed that targeting SPLWs in areas of high socioeconomic deprivation—which was a focus at the very start of the original SPLW programme in Scotland—was key to their effectiveness. Concerns about funding inadequacies posed substantial challenges, and the short-term nature of funding was a concern that was raised by interviewees in both England and Scotland. All strategic leads interviewed believed that securing long-term mainstream funding was crucial to the continuation of the SPLW programme, as was adequate funding of VCSE organisations.

## Discussion

To our knowledge, this study is the largest qualitative study on SPLWs to date, reporting from a range of stakeholders’ perspectives. Our findings highlight that SPLWs are increasingly supporting patients with the fundamental social determinants of health, especially in socioeconomically deprived areas; such patients commonly also have multiple, complex health issues, including mental illnesses. SPLWs need time and empathy to build therapeutic relationships with such patients, but because they are neither mental health workers nor case managers, they need to set clear boundaries around the help they provide. Integration within general practice teams varies, influenced by differing employment models. Models whereby SPLWs are integrated in one or two general practices, with support from their employer and SPLW network, appear to be the most successful. However, in both countries, the sustainability of the SPLW role is threatened by unstable funding, increasing service demands due to ongoing economic austerity and worsening access to statutory and non-statutory services.

In line with our findings, a meta-ethnography of 21 studies conducted by Turk and colleagues^25^ found that SPLWs’ experiences were shaped by tensions between policy expectations and the real-world structural and material realities of their work, affecting both the effectiveness and sustainability of their role. The authors recommended clearer delineation roles, better training for SPLWs, stronger infrastructure, and sustained support for socioeconomically disadvantaged groups, stressing that SPLWs cannot substitute for wider social policies to tackle health inequities, which is increasingly acknowledged internationally.

This focus on health inequities reflects the impact of post-2008 austerity cuts in public expenditure in the UK, which has created systemic challenges that SPLWs must navigate. Since 2010, these cuts have disproportionately affected socially and economically disadvantaged groups^26^ and reduced the capacity of local authorities,^27^ mental health services,^28^ and state benefits^29^ to sufficiently support patients. These austerity measures have taken place against a backdrop of growing patient complexity, especially in socioeconomically deprived areas.^1^^,^^5^^,^^26^ Given these challenges, there is a growing emphasis within UK health policy research and advisory bodies that addressing the core social determinants of health must be central to effectively supporting patients’ health. NHS investment, cross-sector collaboration, and rigorous evaluation are needed to establish what works, for whom, and in what context. Earlier quantitative work as part of our multimethods study suggested that, in England, the roll-out of SPLWs has not been sufficiently targeted at areas of highest need.^30^ With the increased focus on supporting social determinants, areas with high levels of socioeconomic deprivation might require additional resources to recruit and retain SPLWs to tackle health inequalities and better support population needs.

Our research highlighted that the SPLW role works best when fully integrated into general practices and with supportive SPLW networks, in line with recent findings that SPLW integration in general practices varied from “bolted on” to fully “belonging”.^31^ In our study, SPLWs stressed that taking time to ensure that patients feel heard and showing empathy were central to building therapeutic relationships, especially in patients with complex needs, aligning with recent research.^32^ Moudatsou and colleagues^33^ highlight that empathy is essential for building effective therapeutic relationships but that sustaining empathy requires training and support to manage the associated pressures. Similarly, Westlake and colleagues^34^ found that SPLWs frequently engage in continuing contact with patients with complex needs while waiting to access other services. Although this approach provided vital intermediate support and eased pressures on primary care, insufficient training and supervision, and a lack of structural support could lead to patient dependency and burnout and attrition of SPLWs. In related work, Tierney and colleagues^35^ reported SPLW burnout and turnover driven by mismatched job expectations, low self-efficacy, and insufficient support.

A key strength of this study was the large number and range of stakeholders interviewed. We used a systematic, structured approach to the thematic analysis, led by a highly experienced qualitative researcher (ED), who coordinated the analysis conducted by the researchers involved. Findings and interpretations were regularly discussed within a large, multidisciplinary team of researchers.

Our study also has several limitations. For instance, a limitation of the study was that patients were nominated by SPLWs, which might have introduced selection bias, despite our requests to include patients who needed more support to engage with SPLW services. Additionally, despite efforts to purposively sample for ethnic diversity, there were only two patient interviewees who were of ethnicities other than White. Additionally, due to the large number of interviews conducted and large volume of data, we did not analyse the data specifically for gender differences—which could be a useful focus for future work—or look specifically for minor themes or diverse cases in detail. The lack of data collection on demographic characteristics for the other stakeholders was also a potential limitation of our study.

As with all qualitative research, we cannot claim our findings to be generalisable or transferable to other settings. For example, how SPLWs function in other regions of the UK, such as very remote and rural settings, cannot be ascertained from the present study. Additionally, the apparent differences between regions reported by SPLWs in the present study cannot be regarded as necessarily representative, given the small number of participants interviewed per region—for this reason, we have not identified the regions in question. However, this study is part of a large programme of work involving large national primary care datasets, and synthesis and integration of the findings from the qualitative and quantitative work will be triangulated at a later date.

To secure the long-term sustainability of SPLWs, robust and stable funding models must be established to mitigate the volatility currently affecting SPLW roles. Additionally, there should be strategic efforts to address the root causes of health inequalities—beyond the scope of SPLWs—to ensure comprehensive support for communities in need. Further work is required on clearer role definitions. Enhancing the training and career development pathways for SPLWs could help improve job satisfaction and retention rates, together with establishing boundaries and standard operating procedures to manage risks effectively. Our study emphasises that SPLWs are increasingly supporting issues related to the social determinants of health, especially in socioeconomically deprived areas. Much of this activity is not captured routinely in primary care records. Any existing data are spread across multiple systems and files, making it difficult to document and show the benefits of the support provided by SPLWs. Improved integration of SPLWs into general practice and primary care teams is crucial. This integration should foster a supportive network and facilitate regular communication within general practices. It is essential for SPLWs to receive adequate clinical and general supervision, training, and support, particularly when dealing with patients with complex needs. Although empathy and relationships are key, there should also be an emphasis on maintaining therapeutic boundaries, ensuring that SPLWs are not overburdened with duties outside of their remit.

Integration of SPLWs into the primary care team is also beneficial for patients, especially for those with multiple complex health and social care needs, who already face a wide array of fragmented services across the NHS and social care networks. Education of GPs (including GPs in training) and primary care teams regarding social prescribing and the role, responsibilities, and function of SPLWs is important to ensure the appropriate use of social prescribing and provision of joined up care for patients.

In summary, our findings indicate that rather than simply linking patients to VCSE resources, SPLWs are dealing with patients with highly complex needs, including the basic social determinants of health, especially in those living in socioeconomically deprived areas. If this aspect of the SPLW role is to continue to expand, this change in scope needs to be made explicit during recruitment and addressed in training and ongoing support. Further rigorous research on the SPLW role is necessary to delineate what works and for whom and in what circumstances in social prescribing, considering the complex systems within which SPLWs operate. Consistent data collection methods are needed to establish a clearer evidence base on the effectiveness of SPLWs as their roll-out continues in the UK. Additionally, there is a need to explore the long-term effect that SPLWs might have on health and their role in prevention efforts across different settings and contexts. Rigorous quantitative evaluation is also required to show the effectiveness and cost-effectiveness of SPLWs.

## Data sharing

The data are not publicly available as they contain information that could compromise the anonymity of research participants. Secondary use of interview transcripts is possible but must be accompanied by a separate ethical review to ensure the anonymity of our study participants and maintain the ethical standards adhered to within this project. Transcripts are available on request from the corresponding author.

## Declaration of interests

We declare no competing interests.
