# Supplementary material for: Embedding social prescribing in primary care in England and Scotland: a qualitative study of experiences, roles, challenges, and sustainability
Source: Lancet Prim Care. 2026 Apr;2(4):None. doi: 10.1016/j.lanprc.2026.100128 (PMC13154165; doi:10.1016/j.lanprc.2026.100128)
Supplement: Supplementary appendix [file mmc1.pdf]

THE LANCET
Primary Care

Supplementary appendix

This appendix formed part of the original submission and has been peer reviewed. We post it as supplied by the authors.

Supplement to: Donaghy E, Mercer SW, Brant H, et al. Embedding social prescribing in primary care in England and Scotland: a qualitative study of experiences, roles, challenges, and sustainability. *Lancet Prim Care* 2026. https://doi.org/10.1016/j.lanprc.2026.100128

Appendix

Table of contents

Page Number	Content
2	Appendix Box 1. Region and case site details
3,4	Appendix Table 1. Participant recruitment by region and case study site
5	Appendix Table 2. Issues addressed with stakeholder interviewee
6-8	Coding tree
9	Reflexivity statement
10	Appendix Table 3. Stakeholders approached, refused, and reason for refusal
11	Appendix Table 4. SPLWs length of time in post
12	Appendix Table 5. Referring professionals' details
13-22	Additional quotes

Appendix Box 1. Region and case site details

North East & North Cumbria region

A predominantly urban, post-industrial region in northern England, with significant rural and remote areas in North Cumbria. The region experiences high socio-economic deprivation and health inequality, with marked variation across urban, coastal, and rural communities.

Case study site 1

A metropolitan borough in North East England with a predominantly urban population and relatively high socio-economic deprivation, alongside marked neighbourhood inequalities.

Case study site 2

Predominantly urban city with a young, diverse population and significant socio-economic inequalities, including areas of high deprivation and affluence.

Case study site 3

Predominantly urban metropolitan area alongside a sparsely populated rural areas, reflecting contrasting demographics and marked socio-economic inequalities across urban and rural settings.

West of England region

A mix of urban and peri-urban settlements. Includes areas of strong economic performance and population growth alongside marked socio-economic inequalities, with substantial variation in deprivation and health outcomes between neighbourhoods.

Case study site 1

A predominantly rural area with several urban centres, an older and less ethnically diverse population, and marked socio-economic inequalities despite generally favourable economic conditions.

Case study site 2

A large town with a growing, relatively young and ethnically diverse population, and significant socio-economic inequalities despite favourable employment and economic indicators.

Case study site 3

Large city with unitary authority status, characterised by a young, ethnically diverse population and marked socio-economic inequalities despite strong overall economic performance.

Lothian region

Predominantly urban region in south-east Scotland, centred on a major city with surrounding suburban and semi-rural areas, and marked socio-economic inequalities despite overall strong economic performance.

1 Case study site

Urban city characterised by high overall affluence alongside pronounced socio-economic inequalities, with distinct pockets of deprivation within otherwise prosperous neighbourhoods.

Greater Glasgow & Clyde region

Predominantly urban, post-industrial region in west-central Scotland with high socio-economic deprivation and health inequality, and substantial variation across inner-city, peripheral, and affluent suburban areas.

1 Case study site

Post-industrial Urban city. High levels of socio-economic deprivation, alongside pronounced inequalities between neighbourhoods, areas of severe deprivation existing alongside more affluent communities.

Appendix Table 1. Participant recruitment by region and case study site

Region	Case study site	Number of participants recruited	Local PIs responsible for recruitment
West of England	Site 1	**Total = 18**	ME & HB
West of England	Site 2	**Total = 12**	ME & HB
West of England	Site 3	**Total = 18**	ME & HB
		**Number of individual participants recruited in West of England**<br><br>SPLWs (14)<br>Service user (9)<br>Referring professional (9)<br>VCSE employing/hosting SPLWs (4)<br>VCSE not hosting/employing SPLWs (6)<br>Strategic lead (6)	**Total number of participants recruited in West of England**<br>**48**
**Region**	**Case study site**	**Participants**	**Local PIs responsible for recruitment**
North East & North Cumbria	Site 1	**Total = 8**	MS
North East & North Cumbria	Site 2	**Total = 16**	MS
North East & North Cumbria	Site 3	**Total = 15**	MS
		**Number of individual participants recruited in North East & North Cumbria**<br><br>SPLWs (12)<br>Service user (9)<br>Referring professional (9)<br>VCSE employing/hosting SPLWs (2)<br>VCSE not hosting/employing SPLWs (2)<br>Strategic lead (5)	**Total number of participants recruited in North East & North Cumbria**<br><br>**39**
**Region**	**Case study site**	**Participants**	**Local PIs responsible for recruitment**

Lothian	1 case study site	**Total = 22**	ED
		**Number of individual participants recruited in Lothian**<br><br>SPLW (5)<br>Referring professional (5)<br>Service user (5)<br>VCSE employing/hosting SPLWs (3)<br>VCSE not hosting/employing SPLWs (2)<br>Strategic lead (2)	**Total number of participants recruited in Lothian**<br><br>**22**
**Region**	**Case study site**	**Participants**	**Local PIs responsible for recruitment**
Greater Glasgow & Clyde	1 case study site	**Total = 21**	HC
		**Number of individual participants recruited in Greater Glasgow & Clyde**<br><br>SPLW (5)<br>Service user (5)<br>Referring professional (5)<br>VCSE employing/hosting SPLWs (1)<br>VCSE not hosting/employing SPLWs (4)<br>Strategic lead (1)	**Total number of participants recruited in Greater Glasgow & Clyde**<br><br>**21**
		**Overall participant recruitment by stakeholder group**<br><br>**SPLWs -36**<br>**Service users-28**<br>**Referring professional -28**<br>**VCSEs -24**<br>**Strategic lead -14**	**Overall participant total 130**

Appendix Table 2. Issues addressed with stakeholder interviewee

Interviewee Group	Key issues addressed in semi-structured interviews
Social Prescribing Link Workers [SPLWs]	Background and training; Working with GP surgeries; The referral process; Initial meeting & engaging with service users; Audit-recording of referrals and reviewing outcomes; Outcome measures used; Caseloads; Appropriateness of referrals; Relationship with key staff in GP practices and VCSEs; Autonomy, Competence and Relatedness; Management and supervision; Key support mechanisms; Barriers & facilitators to fulfilling the SPLW role; Future sustainability of SPLWs.
Service users	General social and clinical background; Key issues needing support with; Experience of referral process; Initial thoughts on seeing a SPLW; Initial experience of first meeting with a SPLW; Views on support offered; Experience of being signposted to VCSEs; Follow up and engagement with SPLW and primary care referrer; Autonomy, Competence and Relatedness; Overall impact to service user on seeing a SPLW; Reflections on engaging with SPLW.
Referring professionals	Professional background and demographic details of GP practice; Practice background on getting involved with SPLWs; Personal and practice understanding of social prescribing and SPLWs; Integration of SPLWs with GP practice staff; Experience of working with SPLWs; Identifying and referring patients to SPLWs; Initial referral process; Patients' initial views on idea of seeing SPLW; Discussing SPLW referrals; Assessing service user outcomes after engaging with SPLW; Barriers and facilitators to successful outcomes; views on Autonomy, Competence and Relatedness; Impact of SPLWs on service users; Impact of SPLWs on general practice.
VCSE leads that host/employ SPLWs	Type of support and activities VCSE offers to service users; Background to VCSEs originally hosting/employing SPLWs; Management arrangements between VCSE and individual SPLW; VCSEs relationship with allocated GP practices; Relationship between SPLW and wider VCSE groups; Target population; Monitoring service user engagement and outcomes; Barriers and facilitators to successful service user outcomes; Funding and sustainability of SPLW services; Funding and sustainability of individual VCSE.
VCSEs that don't host/employ SPLWs but take service users signposted to them	Type of support VCSE offers to service users; Target population; Views on SPLW service; How VCSE engages with SPLWs; How VCSE engages with service users signposted to them by SPLWs; VCSE capacity to support service users signposted by SPLWs; Barriers and facilitators to successful service user outcomes; Funding and sustainability of individual VCSE. Views on future of SPLW services.
Strategic leaders involved with SPLW services	Role in relation to SPLWs and what it involves; Background to how SPLW services developed in area; England specific questions on key policy developments around SPLW service development e.g., 2019 long-term plan, ARRS; Scotland specific questions on key policy developments in SPLW service development e.g., 2017 early adopters' schemes and inclusion in Scottish 2018 GP contract; Where does social prescribing and SPLW fit in current primary care landscape; Impact of SPLWs on service users; Impact of SPLWs on general practice; Funding and future sustainability of SPLW services.

Appendix: Coding Tree

SU= Service User; **SPLW**= Social Prescribing Link Worker; **RP**= Referring Professional; **VCSE**= Voluntary, Community and Social Enterprise

Social prescribing link worker (SPLW) coding

This coding tree outlines the coding used to examine social prescribing link workers' (SPLWs) perspectives on their role and practice within primary care. It captures training and professional background, day-to-day work within GP surgeries, referral and engagement processes with service users, the therapeutic encounter and the challenges associated with service user needs and social deprivation. This coding also reflects organisational support, workload and sustainability issues, relationships with VCSEs, and is informed by self-determination theory and candidacy concepts to support interpretation of practice and delivery conditions.

Background and Training

- Previous role/s [and how this experience and previous training help fulfil SPLW role]
- Personal attributes/skills/characteristics that are necessary for SPLW role
- Quality, nature and Impact of SPLW training [who provides it & pays for it?]
- Clinical Supervision
- Current Employer
- Length of time in SPLW role

SPLWs and working in/with GP surgeries

Experiences and challenges of working as SPLWs in/with GP surgeries
Number of GP surgeries covered
Surgery staff awareness, value and understanding of SP and SPLW role
Promoting ongoing awareness of SPLW in GP surgeries
Each GP surgery [and GPs] are different
SPLW integration and relationships with GP surgery staff
SPLW room allocation in GP surgeries

Receiving and processing referrals

- Referral source
- Referral systems used [e.g., Elemental, System One, email, paper, self-referrals]
- Efficiency of referral system
- Processing referrals
- Appropriateness of referrals to SPLWs
- Recording/monitoring data on referrals to SPLWs
- Formal/informal feedback between SPLWs and surgery staff who refer
- First contact – how made and time between receiving referral and first contact with SU

First full discussion meeting with service user

First full formal discussion meeting with service user
Where first full meeting occurs and how responsive SU is
SU uncertainty over SPLW role
Importance of having extended time with SUs at first full discussion meeting
Importance of co-production-identifying SU priorities and next steps
First full discussion with a SU can uncover unmet needs

Range of SPLW provision Initially offered to SU following first full discussion
Follow up arrangements after first full discussion
Importance of transparency in working with SUs
Accompanying SUs to attend community groups
Recording SU VCSE engagement/feedback from VCSEs

Building on the initial first full discussion meeting with service users

- Importance of building trust and rapport with SUs
- SU reservations about engaging with a SPLW or engaging with community groups
- Harder to reach service users

Service user issues

- Growth in social isolation and mental health issues
- Addictions
- Impact of cost-of-living crisis
- Growth in fuel/food poverty
- Benefits/welfare rights
- Growth in housing issues
- Burden of caring responsibilities
- Neurological issues [ADHD]

SU Challenges particularly associated with social deprivation

- Service user trauma [ACE, ATE]
- Limitations of what SPLWs can deliver given scale of SUs problems
- Poor IT literacy skills
- Poor English literacy skills
- Poor general organisational skills

Barriers /facilitators to successful link worker intervention

- Barriers to successful link worker interventions
- Facilitators to successful link worker interventions
- SPLW Long-term sustainability/Longevity

Number of sessions with SUs

- Are sessions rationed or open-ended

Measuring and recording service user outcomes

- Measuring and recording SU outcomes
- Well-being tools used
- SPLW issues with using wellbeing tools

Link worker caseloads

- Average caseload size, Waiting list - Cap to list – ever close list
- Managing caseloads – within SPLW team/across GP practices
- Disengagement from SU/closing cases

Formal SPLW line-management support/supervision

- Formal line management support
- Addressing organisational/management issues and how this works
- Importance of city-wide SPLW leadership network

- Importance/Access to peer support
- SPLW role can be isolating
- Need for more embedded support network and shared learning for SPLWs

Psychological support for SPLWs

- Psychological toll of being a link worker- compassion fatigue/trauma
- Importance of support from clinical psychologist

SPLW terms and conditions issues

- Different pay scales and terms and conditions across VCSEs/PCNs hosting SPLWs
- Opportunities for career progression

VCSE capacity to support SPSPLW

- Lack of/reduced capacity of VCSEs to meet demand
- How do SPLWs source appropriate VCSE services

Building/developing/maintaining/updating VCSE links

Continually developing/maintaining/updating VCSE links

- Feedback about SUs engaging with community groups

Appendix: Reflexivity Statement

The reflexive inductive thematic analysis was conducted by authors ED, HC, HB, ME, YH and MS. Although analysts SWM, CS, C O'D and PW were not involved in the interviewing, they are experienced qualitative and mixed methods researchers with substantial expertise in primary care and in-depth knowledge of link worker roles and service delivery contexts. These analysts brought prior professional and research experience relevant to the study topic and remained reflexively attentive to how these perspectives could shape data interpretation. Reflexivity was maintained through monthly team data analysis discussions, comparison of coding, and iterative reflection during theme development. The involvement of those analysts not engaged directly in data collection also supported critical distance and alternative interpretations.

Appendix Table 3. Stakeholders approached, refused, and reason for refusal

	**Number Invited**	**Number Refused**	**Reason**	**Number Interviewed**
SPLWs	40	4	2 didn't want to take part; 2 no reply	36
Patients	34	6	2 didn't want to take part; 1 was too busy; 3 no reply	28
RPs	36	8	3 too busy; 1 unavailable before the deadline; 4 no reply	28
VCSEs	24	10	1 unavailable; 9 no reply	14
Strategic leads	16	2	2 no reply	14
**Total**	**160**	**30**		**130**

SPLWs = Social prescribing Link Workers; RPs = Referring Professionals; VCSEs = Voluntary, Community, and Social Enterprise Organisations

Appendix Table 4. SPLWs length of time in post (n=36)

Years	Number
1 year or less	11
2 years	10
3 years	5
4 years	4
5 years	4
6 years	1
7 years	1

Appendix Table 5. Referring professionals' details (n=28)

Professional post	Number
GPs	18
Pharmacist	2
Nurse	2
Care co-ordinator	2
Mental health worker	1
Counsellor	1
Health visitor	1
Teacher	1

Additional quotes to key themes

BOX 1: Key to stakeholder interviewee abbreviations in presented quotes

NENC = North East & North Cumbria (3 case study sites)
WE = West of England (3 case study sites)
GG&C = Greater Glasgow and Clyde (1 case study site)
L = Lothian (1 case study site)

CS = Case study site (with associated case study site number)

SU = service user (patient)
SPLW = social prescribing link worker
RP = referring professional
VCSE hosting = VCSE hosting/employing SPLWs
VCSE non-hosting = VCSEs not hosting/employing SPLWs but take service user referrals
SL = strategic lead with SPLW services

Theme One: Background and motivation to become a link worker

SPLWs come from a wide-ranging background and have a broad range of skills in community support, mental health, housing, education, and holistic therapies. They are experienced in 1-2-1 support, signposting, tackling stigma, building resilience, and empowering individuals through prevention-focused work. With a strong commitment to social justice and helping people, they believe they are connecting service users to vital services and supporting better long-term health outcomes.

> *"I had been semi-retired. I left the local authority. I'd worked in housing, and the OT service. In the latter part of my career, I went into management, but I didn't enjoy it in the same way as I enjoyed working frontline and seeing people on a daily basis."* [NENC_CS1_SPLW4]

> *My previous role was managing an information service. It was effectively light touch link working. Providing a signposting service to people seeking information about mental health services, about a recent or historical diagnosis. It was one or two contacts with people. That gave me a really good foundation about the service landscape in the city. It helped me build skills around having difficult conversations. Building my own personal resilience. It's a really good foundation for this career.* (L_SPLW3)

> *I've got a degree and a master's degree. I've worked in supported accommodation within homeless populations. That was my first insight into health and social care and social issues. I could see how the trajectory of youth, if there was no intervention and prevention, it could affect the trajectory of their adult life. That taught me about health inequalities, social deprivation. I always wanted to be part of the prevention work. I think the links work compliments that quite a bit and hopefully prevents some of the worst health outcomes in the long run.* (GG&C_SPLW1)

"I can work well in this role and the autonomy suits me because I'm overly motivated, I can manage my own time and that sort of thing." (NENC_CS2_ SPLW2)
"I think the reason I like this job is it's not the pay because it's not ideal but just having that [SPLW employing organization] is very good at having that flexibility in your work." (GG&C_SPLW2)

Theme Two: The changing role of the social prescribing link worker

Increasing service user complexity and help with basic socioeconomic needs

Interviewees across all sites reported rising patient complexity, particularly in deprived communities. Service users referred to SPLWs by GP practice staff often face overlapping challenges—chronic illness, poor mental health, childhood and teenage trauma, social isolation, and financial hardship—requiring more time and support than straightforward signposting. Stakeholders noted SPLWs are increasingly filling gaps left by stretched mental health and social services, working beyond their remit as COVID-19 pandemic and the cost-of-living crisis intensify poverty, trauma, and isolation, driving more complex referrals.

Increasing service user complexity

In our area, deprivation and low income, people with complex health needs, a lot people couldn't really access advice because they can't take it in on the phone. My caseload varies from about 35 to 50. About 15-20 are complex cases where there's a lot of input over time. Then there'll be about five of them I've seen for more than a year because their needs are never going to go away and they keep coming back with other issues. [NENC_CS2_SPLW2]

When we first started, SPLWs only worked with people with low level mental health issues. But that changed very quickly, particularly with the COVID-19 pandemic. Now we get quite complex mental health issues. I'm working with a gentleman who was suicidal. One patient I've worked with, the GPs said - you will never be able to discharge her because of her severe and complex needs. And I've had about 60 plus sessions with her. [WE_CS2_SPLW1]

*When there's pressure on mental health services, social services, and the charitable sector, they have pushed case management back to link workers. There have been services who have then used links workers as a primary support. We've really tried to manage that. I've had letters from psychiatrists saying '*I'm discharging this person to their ongoing support with the links worker*'. I'm like, absolutely, no you're not!* [GG&C_CS1_RP2]

I've got a chap on my caseload who he has probably been referred to me ten times in six years. We have not found the right support. It doesn't exist. Social work is broken. He needs long-term support, which doesn't exist…..One example of a woman recently who had just left an abusive relationship, was in a temporary accommodation and was really withdrawn. To me, she wasn't appropriate for the [SPLW] *service because she wasn't ready to be being linked. She needed trauma support.* [L_ CS1_SPLW4]

I work with service users who are experiencing a wide variety of personal difficulties they need emotional and practical support and often have a diagnosis of mental health or chronic physical illness. A lot of people have had difficult situations with [statutory] *services where*

they've felt incredibly let down. So, it's building up that trust and a working relationship with them and doing some work around that and managing anxiety [NENC_CS3__SPLW3]

Increasing help with basic socioeconomic needs

SPLWs reported that their role is increasingly dominated by core socio-economic issues such as food insecurity, debt, poor housing and the mental health impacts of poverty. Many patients present with health concerns, but underlying struggles with income, heating and basic needs quickly emerge, showing how systemic inequalities are driving demand for such support.

> *Unfortunately, the main thing that the link workers are dealing with now, is the food insecurity, financial difficulties and inadequate housing- - and poor mental health - which, obviously, impacts on every single one of those other issues. So, trying to deal with those very practical issues*. [L_ CS1_VCSE 3 _Hosting]

> *Patients would've come because they've had frequent coughs or colds and they would say the house is damp and I've got no money for heating my flat over the winter. So, those tend to be the things. So, there will be potentially a medical reason for them originally coming, but as you peel back the layers you also realise that these are the struggles going on within their lives.* [WE_CS1_RP2]

> *It doesn't take a genius to work out that people are presenting with complex, deeply rooted, social-economic problems that spread into the health effects of that…..The countless number of people that refer- are for practical benefits around debt, welfare, benefits, income, financial insecurity-the commonest immediate issues of - what matters to you?* [NENC_CS1_RP3]

*Inequalities is driven by social factors. And the social factors are the wider determinants of health. We run a Food Club. We're trying to spread that across the whole area…..They might not be able to afford to heat their homes, but actually you can move money from food to heating, if you get food cheaply. [*WE_CS3 _VCSE1 _Hosting]

Where I am, it's a deprived area, so there's financial stress. The top issues are mental health, social isolation and basic core needs. The cost-of-living crisis, fuel, food poverty. And housing. The council housing service in the city is just woefully inadequate …. People's core needs not being met. It's systemic… We're down the stream, hauling the bodies out, but ultimately you need to go upstream and stop the bodies falling in in the first place. Because there's only so much we can do as SPLWs. [L_CS1_SPLW3]

Accessing statutory services

SPLWs highlighted growing difficulties in accessing statutory services: councils, benefits systems, mental health services are overstretched, under-resourced and slow to respond, leaving people without timely support. Rising thresholds and long waits mean many are excluded from housing, mental health and social care, while complex systems and digital access issues further marginalise vulnerable patients. As a result, SPLWs spend significant time advocating on behalf of service users who feel ignored or dismissed.

With the council, they're just overwhelmed…..The housing officers, they don't always get back to you, don't respond to emails……… The biggest problem I have is that a lot of the

agencies we refer to are struggling. They don't have the finances, the time, or the staff to deal with the huge problems that are out there. [WE_CS3_SPLW2]

We do a lot of work on behalf of patients to have their voice heard or pushing things so that people get calls back [from statutory services]. *There're a lot of tardiness and people don't get called back or they feel dismissed. There's definitely a lot of advocacy work, acting on behalf of patients, where they feel that the systems are not listening to them or responding to them.* [L_CS1_SPLW2]

Longer appointments are needed so people can navigate [statutory] *systems. Because systems at the moment are so complex and all we're doing is marginalizing people even further through digital exclusion. The point of access has been quite challenging. There's a longer waiting list. I was writing a report today on the gaps and challenges. It's still the same things that we're going on about - the gaps in* [statutory] *services - mental health, housing.* [GG&C_CS1_VCSE 1 _Hosting]

There are pressures across the entire system, including secondary care, social care., et cetera. I think the global overall picture is one of greater complexity of needs- that's because the thresholds of many statutory services are higher and higher. Lots more people are being bounced out and there's a clearing of the decks in some specialist services. So, we're seeing more people who are notably unwell and the nature of that unwellness is more complex [NENC_CS3_VCSE1 lead not hosting SPLW]

Theme Three: The key role of the therapeutic relationship – time, empathy and trust

SPLWs consistently described active listening as central to their role, providing marginalised service users with validation, empathy, and a non-judgemental, trusted, space to be heard. Many reported that simply being listened to improved wellbeing for many service users, especially given long waits for statutory mental health services and limited time with GPs. While SPLWs acknowledged they are not a substitute for talking therapies, such as counsellors they stressed the value of offering confidential listening support, helping people feel they matter, validated.

People are voiceless, completely marginalised, often ignored and not heard. So, your job is the listening. It's just hearing people. That can be powerful for people. Feeling that they matter and what they've got to say matters. So that's really at the core of the job……A lot of it is listening, not judging, being completely impartial. I think people respond to that genuineness and listening' You do a lot of listening. [NENC_CS1_SPLW2]

I feel like we're filling a gap, that go-between emotionally supporting people who are having to wait a ridiculous amount of time [for statutory service support]. *Often, it's listening at first. They haven't had a chance just to offload and explain everything to someone external that's not their family or friendship group. That's quite nice for them. They know they're not being judged and it's confidential. A lot of it's just offloading at first and then we start to unpick it together.* [WE_CS3_SPLW2]

Because there is a one year waiting list for talking therapies, it's a natural side effect that some people's high need, they just want to use us as talking therapies. These people need to be heard, of course, because GPs don't have time to hear them. And only by hearing

them can we discern what matters to them, yet not to take it into talking therapies realm. [WE_CS3_SPLW3]

Most people will leave the appointment and they will be like- I feel much better after having that conversation. I think people are always going to need that social support. [NENC_CS3_SPLW3]

*Having really good strong active listening skills is very important. Showing somebody that you're not just hearing what you're saying, you're actually listening to them. Not being judgemental. The amount of people that say to me "*do you know what - nobody's ever actually sat and listened to me like that*". It's validating for people to know that it's okay to come in and talk. That's really valuable for people.* [L_CS1_SPLW3]

I find one of the big one's [for service users] *is that they need someone to talk to about their issue. That's not going to solve the issue but sometimes it's just that they haven't had anyone to talk to about this or they just want to be heard.* [GG&C_CS1_SPLW2]

Theme Four: Benefits of SPLWs

Benefits to Service Users

Participants described improvements in confidence, access to supportive networks, and engagement in community activities. Examples included families with children who have special needs, practical assistance with everyday practical issues, even stretching to providing holiday support. More broadly, SPLWs were seen as providing both emotional help and practical guidance, helping individuals to reduce isolation, manage challenges, and build confidence.

[What difference did link worker make?] *Massive. Without that I wouldn't be here. I'd still be in the world. I just wouldn't be here in this place. I'd just be sitting in the house doing nothing and that's not good. I became almost agoraphobic. I didn't want to go out, or I wasn't going out and then I couldn't go out. They've just given us so much freedom just to express myself. All the extra help is fantastic but just talking about it is good enough for me to start with.* [NENC_CS3_SU1]

*All I know is that she [*SPLW] *has made a massive, massive difference. I can't really explain how big that difference is to our family. Through the use of that [SPLW] service. Getting holiday support when you have children with special needs. I can't believe a service like this exists. It's incredible. An incredible difference to special needs children, massive. She's changed our lives for the next twelve months with that holiday support.* [WE_CS1_SU2]

The links worker spoke to me about the carers support group. It meets every month. It's other parents experiencing what I'm experiencing [child with disability]. *It was a good support network. The girls at the group would be like, "oh have you tried this, have you tried that". Other mums just bouncing ideas off each other. If it hadn't been for a links worker, I would probably be trying to do this all on my own. Not opening up about what was happening at home, not opening up about how I struggled with my child, not knowing where to go, who to speak to.* [GG&C_CS1_SU1]

I've struggled with my mental health for quite a while and I've never had this experience with somebody doing what the link worker has done. Perhaps if I'd had this opportunity before, I would be in a different place by now. I'm very grateful to have had that opportunity. What did I get? Confidence - that I could go along to groups myself. So, confidence and enjoyment. [L_CS1_SU3]

Benefits to GPs

GPs highlight that SPLWs play a crucial role in meeting patients' non-medical needs, and easing GP workload. They provide patients with the right kind of support, time, and expertise that GPs often cannot offer, leading to fewer repeat visits for non-medical issues. While SPLWs can unearth unmet needs, their involvement ultimately benefits both patients—through more tailored support—and GPs, by alleviating the burden of challenging, non-clinical cases. Concerns remain that if SPLW funding were withdrawn, patients would return to general practice for this support, increasing pressure on already stretched services.

[with the SPLW] *The total volume of appointments for patients who we were seeing a lot is down. But the other thing is that often some of those appointments were some of the most challenging. It was often things that we thought* [as GPs]- *I know this is affecting your quality of life. I just know I'm not the best equipped to help. I don't have the time to listen as well as I would like to. And having a* [SPLW] *service that both knows more of what could be helped with - and has more time - is absolutely invaluable.* [L_CS1_RP2]

[after seeing the SPLW] *I think service users are less likely to come back to the practice for a non-medical cause. That definitely it makes a difference. We would probably be quite lost if we didn't have link workers. We'd feel the burden of work they've been doing back onto us……The biggest impact* [of SPLWs] *on patients is that they actually get the right kind of support. As GPs, we don't have (a) the time, or (b) the knowledge or expertise* [that SPLW have]. *Having the link workers there is just really helpful.* [NENC_CS1_RP2]

Obviously, a successful outcome is they come to see us [GPs] *less often. They've got somebody* [SPLW] *more useful working with them, so that's good for the patient - someone's helping them more. But it's good for the GP because we're not seeing people who are really not appropriate.* [NENC_CS3_RP2]

Recently, the funding has changed and there has been a lot of concerns, particularly amongst the GPs. If link workers were to be withdrawn from general practice, what would happen to the patients who need that low level support and how would we signpost these patients? Those patients will start to come back to us [GPs] *and increase our workload. They* [SPLWs] *have been really good at reducing the workload of the low-level stuff that's non-medical.* [WE_CS1_RP]

Theme Five: Organisation and management of SPLWs

Retention

Retention was reportedly a problem, related to factors such as burn-out, low pay, lack of job security, lack of career structure/progression, insufficient training, support and clinical supervision, stress and isolation - especially those working across several GP practices or where they didn't feel part of the GP practice team/s.

> *There's no standardisation yet so staff aren't getting really important things like clinical supervision, which is important for our mental wellbeing as well as client care. Considering the types of complex cases, we take on. The system isn't where it needs to be and it isn't perfect. But it's a very new role, so it's not managed in the same way as nursing or other professional roles in the NHS yet. I would say the most important thing for link workers for me would be clinical supervision really, to try and stay on top of their own mental wellbeing.* (NENC_CS2_SPLW1)

> *My line manager encouraged me to sign up to the therapy service. Because she was asking about my health, how I am coping with this role. Mine are complex cases and I am getting resources so far, but then sometimes the resources just aren't there. It is hard….I think that's quite stressful at times to manage. We all have our personal lives and the* [SPLW] *role has to be permanent; (non-permanence) adds a bit more stress for me. We also have to get paid more.* (GG&C_SPLW5)

> *The independence in the role can be challenging, the fact that it can be isolating, because you're carrying the whole load of all these patients and all their awful problems. Often, it can feel isolating. (NENC_CS1_SPLW2)*

> *I had a series of really emotional and difficult cases. I was offered, and I did see a counsellor. I just said to my line manager, these have been really demanding, really emotional. It can be very difficult, when people are talking about their past history of sexual abuse. The* [SPLW role] *can be hard. I actually saw this patient; he was feeling suicidal. He'd been cutting himself really badly for the week beforehand. I was with him for three hours, just trying to get support. He was in a really bad way.* (WE_CS3_SPLW2)

> *I was in the job 18 months before I got a supervision. I think in the contract, it said we'd have monthly supervisions and clinical supervisions. Four and a half years in, I have not had a clinical supervision.. At the beginning, this job, it can be very overwhelming and people take it home and they dwell on it at home. Because we see a lot of awful things. People living in squalor, in poverty, people in very ill health. Seeing people's toes drop off, stuff like that….I used to take it home and you'd get upset about it and it's draining. I think for any newcomers, it would impact.* (NENC_CS2_SPLW3)

Working within general practice

Experiences of SPLWs in GP practices varied widely. Some surgeries, particularly those with a strong history and support for the concept of social prescribing, were highly supportive and integrated SPLWs into practice teams, while some remained more medically focused and less engaged. Over time, understanding of the role has generally improved, though SPLWs still need to set clear boundaries and advocate for their remit.

From my experience there's often a few key staff in GP practices that really get it (SPLWs). Often you get a lot of referrals from the key staff who are invested in it…..Some medical staff are a bit more old fashioned in their thinking and like, - no, we're medical, we're here to do medical stuff and aren't so open to exploring different ways of improving people's health….We've invited ourselves along to practice meetings to introduce ourselves. It's not always a very warm reception. (NENC_CS2_SPLW5)

If I'm honest, it does vary across the surgeries [understanding of SPLWs]. *I can only go on my hosted GP practice. I think generally across the PCN, most clinicians have a good understanding of social prescribing. Some of the surgeries appreciate it more than others….I'm not a social worker and there are times when you're treated like that. I have to really lay my boundaries down, certainly with the GPs, and remind them - my remit is- this, this and this.* (GS_CS1_SPLW1)

W*e are very fortunate and I think having that very integrated way of working really helps. Yes, I think they do. But we often talk about - is that referral appropriate…..We do get ones which are effectively supporting adult social care. And I think we do have to remind, on a regular basis, that we are not support workers.* (B2_CS3_SPLW1)

The previous link worker was there seven years, and before that there was two others. Maybe say ten years in total they've had a links worker, so they understand the programme. The lead GP of my practice is very heavily involved. She gets the wider picture. She understands social deprivation and sees the benefits of link workers. (GG&C_SPLW1)

We were one of the original practices that took part, so there's been a link worker here for a long time. In our practice, the GP partners are involved with the Deep End groups [most deprived GP practices]. *The concept of a link worker they all think it's really important. So any GP trainee, medical students, will shadow me. So, they try to spread what the SPLW role is. I take part in MDT meetings as well.* (GG&C_SPLW3)

GP practice staff understanding SPLWs? It's changing all the time. In 2017 when we started, I would have given you a very different answer. Then we had to explain ourselves a lot, and almost justify our presence. We were at the bottom of the pecking order when it came to allocating rooms. One thing that I've learned about working in GP practices is they're all incredibly different. Some have a more holistic understanding of health. Others are more traditionally medicalised. The culture of the surgery has a really big impact on SPLWs. But it's significantly improved over the past seven years. It's not such a novelty now. (L_SPLW3)

Theme Six: Challenges and Sustainability

Risk management

SPLWs can face role ambiguity, often being mistaken for mental health, therapists, or support workers, which risks masking gaps in statutory services. Their remit of connecting patients to community resources can be undermined, particularly in deprived areas, where services are limited or ill-equipped for complex needs such as trauma, addiction, and significant mental health problems. Many SPLWs

reported these challenges have been exacerbated post-COVID-19. SPLWs reported being inappropriately referred highly complex cases, leading to role strain, blurred boundaries, and limited capacity to provide sustained support where other statutory agencies have withdrawn despite service users' ongoing needs.

> *I think that social prescribers still are really not defined in what they are, on what their role is because I think some people think they are mental health support workers…..My concern is they* [SPLWs] *hide gaps* [in statutory services]. *What I say to people is - if you need a support worker, you should get a support worker. If you need a mental health support worker, you should have a mental health support worker. Staff who have relevant training, relevant clinical supervision, and is surrounded by a team that can help them.* [WE_ CS1__SL1]

> *The idea of link work, this was confusing when I started. Because there were different messages around what link work is. So, some people would say, oh, you're just linking your patients to other organisations and they will provide the help. But what you find, certainly in areas of deprivation and low-income, people with complex needs, disabilities, mental health issues, addictions; One, often the services don't exist, and then; Two, if the service exists, it often can't cater to the complex needs of that individual.* [NENC_CS1_SPLW2]

> *A lot of people have quite severe adverse events from childhood, sexual abuse, neglect, physical abuse. I have a lot of people in their in their forties and fifties. All the stuff from childhood starts coming back. Or, because of that abuse, they're using coping strategies that are quite negative - alcohol, drugs- but still have quite severe anxiety, depression, isolation. From COVID, it's got worse….So, I'm trying to learn how to set boundaries and think, this is going too far from what I can actually do.* [GG&C_SPLW3]

> *It was difficult because some GP surgeries were just sending us really chaotic people. They were only thinking about us when they couldn't help this patient. So, they would send people to us that we couldn't really help. They had lots of services already involved, and there was nothing much we could do, except complicate matters.* [NENC_CS2_SPLW3]

> *Usually, people come with complex mental or post-traumatic disorders, or several diagnoses and involvement with mental health services, and they're falling through the gap. Or lots of agencies will have finished working with them and don't want them back. They come to us. That's difficult. Because, I don't know why that support has run out - because their needs have not run out.* [WE_ CS3_SPLW3]

Funding and sustainability

For many stakeholders interviewed, especially VCSE leads and SPLW strategic leads, the biggest challenge for the future of VCSEs and SPLWs is the lack of sustainable funding. Funding is usually short-term, insecure, and often insufficient, creating instability for services and staff, and threatening the effectiveness of future social prescribing services.

> *The biggest thing I do in my role is trying and sustain investment in the voluntary sector, and it is a challenge, lots of our partners are hand to mouth. There's this challenge- do we try to sustain as many projects as possible or do we say, no, these are the strongest 20 or 30 projects. I don't know the best way to go about that because it's a time of reducing resources. It's the sustainability of our community interventions. Because if we haven't got the community interventions social prescribing will not work, it needs the two bits, it needs link workers and it needs community interventions.* [WE_CS3_SL1]

> [What's the biggest challenges VCSEs have in providing SPLW services?] *Commissioning, funding, and changes to contracts. When I started in post,* [named local VCSE] *all three PCNs were using them for their social prescribing contracts; They now only have one. That obviously has an impact on funding streams, losing staff, staff jumping ship because of job security. I think that's probably the biggest challenge for the VCSE partners. The kind of constant worry of future funding*. [WE_CS3_VCSE 1 lead not hosting SPLW]

> *We didn't know until about four weeks ago whether one of our most popular programmes was going to get funded or not. Luckily, it did. It had to be changed slightly, which was fine. You do not know until last minute. That's a lot of stress for staff as well because again you can lose staff through that. People have got mortgages to pay.* [GG&C_VCSE2_not hosting]
